# Physical work demands and expected labor market affiliation (ELMA): Prospective cohort with register-follow-up among 46 169 employees

**DOI:** 10.5271/sjweh.4050

**Published:** 2022-10-29

**Authors:** Jacob Pedersen, Jakob Bue Bjorner, Lars L Andersen

**Affiliations:** 1 National Research Centre for the Working Environment, Copenhagen, Denmark; 2 Department of Public Health, University of Copenhagen, Copenhagen, Denmark.; 3 QualityMetric, Lincoln, RI, USA.

**Keywords:** multi-state, longitudinal, sickness absence, unemployment, work

## Abstract

**Objective:**

This study aimed to estimate the impact of high physical work demands on expected labor market affiliation (ELMA) among men and women of different ages in the general working population.

**Methods:**

After participating in the Danish Work Environment and Health study (2012, 2014, and/or 2016), 46 169 employees were followed for two years in national registers. Using multi-state modeling, taking all day-to-day transition probabilities of labor market affiliation into account (work, unemployment, sickness absence, temporary out, and permanently out), and performing multilevel adjustment, we estimated the prospective association between physical work demands (ergonomic index including 7 factors) and ELMA.

**Results:**

During 104 896 person-years of follow-up, we identified of 439 045 transitions. Using low physical work demands as reference, higher physical work demands were associated with fewer days of active work (2–35 days) during 730 days of follow-up, and more days of sickness absence (4–26 days) and unemployment (ranging 1-9 days) among men and women of aged 40–49 and 50–64 years. Among men and women aged 18–39 years, high physical work demands only had minor and inconsistent impact on ELMA.

**Conclusions:**

Analyzing multiple and highly detailed patterns of transition probabilities concerning labor market affiliation, we showed that reducing physical work demands is likely to increase the active working time and prevent high societal cost of sickness absence and unemployment, especially among middle-aged and older workers.

Several job groups are characterized by high physical work demands, eg, painters, bricklayers, masons, carpenters, cleaners, industrial labor, manufacturing labor, and service work (1). Even with technological advances, many job groups will likely continue having high physical work demands in the future. Additionally, the 2021 National Health Profile in Denmark shows that the prevalence of disc herniation or other back diseases increases from 6.6% for men and 7.4% for women aged 25–34 years to 21.5% for men and 20.2% for women age 55–64 years, respectively. The prevalence of osteoarthritis is even higher, especially among women, where it increases from 23.2% at 45–54 years to 41.0% at 55–64 years (2).

Previous studies have shown that high physical work demands increase the risk of sickness absence (3–6). These studies typically rely on risk assessment of a single outcome – like the probability of a transition from work to sickness absence – while leaving information about other labor market outcomes unattended. However, in several European countries, including the Scandinavian countries, the labor market is quite flexible meaning that individuals are likely to have multiple periods of sickness absence without being fired, and to have recurrent events of unemployment.

Multi-state analysis is an effective way of analyzing the impact on the labor market affiliation when the system is highly flexible and contain multiple states. This study uses the expected labor market affiliation (ELMA) method developed by Pedersen et al (7) for analyzing the impact on labor market affiliation of Danish employees having different levels of physical work demands. The ELMA method relies on multi-state modeling of the labor market system for analyzing multiple transitions and summarizing the effect into expected durations of each state (8–10). In addition, the ELMA method provides the possibility to include variables that may change during follow-up, eg, the individual level of education or civil status, adjustment for multiple variables simultaneously, and weights for making the results representative.

The aim of the present study is to estimate the impact of high physical work demands on ELMA among men and women of different ages in the general Danish working population. The analyses rely on multi-state modeling of the labor market transitions and focus on time in work, sickness absence, and unemployment.

## Methods

### Study design and source population

This longitudinal study uses a linkage of registers and survey data on physical work demands from three successive waves of the Work Environment and Health in Denmark (WEHD) survey conducted in 2012, 2014, and 2016 (11, 12). The survey data was linked to other registers through an encrypted version of the central person register number (13). All WEHD responders, aged 18–64 years, were included and followed in registers for two years from the day they answered the questionnaire.

The WEHD surveys were linked with the following registers, provided by Statistics Denmark: (i) the Danish Labor Market Accountant Register (LMAR), (ii) Register of Work Absences (RoWA), (iii) the Education Register, (iv) Emigration and Immigration Register, and (v) the Death Register. LMAR contains information on all major social benefits payments, including unemployment, sickness absence, disability pension, pension, and all salary payments reported to the tax authorities from 2008 onwards.

RoWA is a linkage of the Absence and Employment Register (FRAN) and the Periods of Absence Register (FRPE), both from Statistics Denmark. FRPE includes date-based information about sickness absence spells from the first day of absence, and FRAN includes date-based employment information of employees with and without sickness absence spells (11). RoWA contains records of both public and private employees. The date-based records of sickness absence spells are complete for all public employees and private companies with >250 employees. RoWA contains a yearly weighted sample from companies with 10–250 employees (14). This means that RoWA covers approximately 37% of all private employees in Denmark (11). RoWA does not include small private companies with <10 employees and these are therefore not included in the present study. Small companies represent a large part of private companies (approximately 260 000 small private companies exist in Denmark) (15). RoWA contains weights for making the private sample representative to all private employees in companies with ≥10 employees. The Education Register contains records of the highest education level completion for all Danes. The Emigration and Immigration Register contains dates on all emigrations and immigrations in Denmark. The Death Register includes dates for all deceased Danes.

The linked data set contains individual and date-based information on labor market affiliation and individual characteristics retained from the surveys.

### Study sample and data preparation

The WEHD data included 67 053 individuals of which 63 912 (95.3%) were eligible for the current study. Receivers of disability pension or retires at the start of the follow-up period (N=2945), individuals aged >64 years at the start of the follow-up (N=195, 28% women), or not found in LMAR (N=1) were excluded.

In RoWA, (i) all records for public employment have the weight one and (ii) all records for private employment have a specialized weight that is constructed based on the sampling probability. RoWA only includes records of individuals in employment, but in this study, the weights were carried forward in LMAR to include periods of unemployment etc, but only until a new employment period.

Records from LMAR that could not be linked to a private or public employment in RoWA were excluded (~7%. 0.6 million records). Similarly, records of private employments without a weight (9%), and public employment period with a specialized weight (0.1%) were excluded.

The final sample was divided into six subsamples according to gender and age-range at the start of the follow-up period (18–39, 40–49, and 50–64 years), prioritizing clearly defined age-intervals over an even number of individuals in each age category. Of the N=46 169 individuals, 78% answered one of the three waves of questionnaires, 8% two questionnaires, and 14% answered all three waves of questionnaires – totalling 62 677 follow-up periods.

### Physical work demand

Physical work demands were measured through an ergonomic index, which was constructed by seven questions (please see supplementary material, www.sjweh.fi/article/4050, A). For individuals answering all seven questions, an average score was calculated ranging from 0–100. All other individuals were registered by a ‘missing’ category. In accordance with Andersen et al (1), the individual average score was categorized into four categories: low (0–10), moderate (>10–20), high (>20–30), and very high (>30) physical work demands. The ergonomic index has shown to predict the risk of long-term sickness absence using standard Cox-regression (1).

### Covariates and weights

The analysis includes nine covariates previously used in studies about physical occupational exposures and physical health in relation to long-term sickness absence (16–18) and work disability (19, 20). The covariates are associated with adverse health outcomes, possible through selection, eg selection into part time work, or through causation, eg, smoking and sickness absence.

Six variables were included from WEHD: (i) working time arrangement (Part-time: <37 hours/week or full time: ≥37 hours/week); (ii) body mass index (BMI) (<18.0, 18.5–<25.0, 25.0–<29.9, and ≥29.9 kg/m^2^); (iii) smoking (yes: daily and sometimes; no: prior smoker and never); (iv) physical activity “How much time on average do you use on each of the following physical activities in the last year?” as “exercise, heavy gardening or fast walking / cycling where you sweat and getting short of breath?” with answers dichotomized as (yes: <2, 2–4 and >4 hours/week; no: “Does not practice this activity” and missing); (v) disease treatment – in terms of a dichotomy variable indicating if the individual has had treatment for one of the following diseases (no/yes): depression, asthma, diabetes, atherosclerosis or blood clot in the heart, blood clot in the brain (cerebral hemorrhage), cancer, back disease, migraine, or other long-term disease; (vi) symptoms of depression, defined by the individual Major Depression Index (MDI) score (depressive symptoms: ≥21; no depressive symptoms: <21) (21); (vii) employment sector (private/public) variable was obtained from FRAN; (viii) highest accomplished education (low/middle/high) variable obtained from the Education Registers; and (ix) “number of survey waves” was constructed to account for the number of WEHD survey waves the individual had attended –1 of 3, 2 of 3, and 3 of 3. Variable (vii–viii) was allowed to change during the follow-up period, while the variables obtained from the surveys (i–vi) could only change if the individual participated in a new survey wave.

### Labor market affiliation

The labor market affiliation was modeled by seven mutually exclusive labor market states based on the longitudinal registrations of LMAR and RoWA. Of the seven states, four are categorized as recurrent states, meaning that multiple individual periods of the same state are possible: (i) *work* reflecting the periods of receiving salary payments and not simultaneously registered as sick-listed; (ii) *sickness absence* for periods when the individual is registered as sick-listed by the employer or receiving sickness absence benefit; (iii) *unemployment* for periods when a person receives social benefit related to unemployment, given the condition that the person is immediately available for work if such opportunity arises; (iv) *temporary out* for periods when an individual is not in the work, sickness absence, or unemployment states but with the possibility of returning to those states. This state contains the time of for example maternity leave, emigration, periods of education, and periods with no registration. The three absorbing states suggests that no further transitions are possible after the first entry; (v) *disability pension* when receiving full or gradually disability retirement pension due to personal disability; (vi) *retirement* due to receiving age retirement pension or the voluntary retirement pension; and (vii) *death* (supplementary material B contains a short introduction to the Danish labor market and social system).

Individuals start the follow-up in any of the four recurrent states.

### Statistical analysis

The study uses the Expected Labor Market Affiliation (ELMA) method developed by Pedersen et al (7), which relies on estimated transition probabilities between the possible states of the multi-state model. The ELMA incorporates both time-dependent variables and time-dependent weights in terms of eg, inverse probability weights. The ELMA uses a non-parametric approach except for the confidence estimation of the expected state duration results.

For each subsample of gender and age groups, we estimated the time-dependent baseline probability for every transition of the multi-state model according to the reference value of the covariates. The transitions probabilities for the non-reference values were estimated by adjusting the corresponding baseline probabilities with estimates derived from Cox proportional hazard regression. The Cox regressions were conducted on the entire multi-state model with the data arranged in a long format (22). Based on the transition probabilities we estimated the state probabilities – expressing the probability of being in one of the seven states from day one and until day 730 (two years).

We summarized the area under each transition probability and state probability curve for each combination of covariates.

Assuming normally distributed area estimates, we produced 500 random resamples and conducted a variance regression model. This was done in order to produce the final estimates of state duration including 95% confidence intervals (CI). All variables, except the ergonomic index variable, were incorporated into the model as inverse probability weights and multiplied by the weights from the employment register.

For light comparison with and control of the ELMA results, a crude estimate of the time spent in each state was made. This was done by summing the time spent in each state during the follow-up period and then dividing by the number of individual follow-up periods.

## Results

Table 1 shows that despite a slight predominance of women (59% women) in the sample, the proportion of individuals in each age group are comparable between the genders (mean age by gender and age group – men: 31.6, 45.3, 56.6 years; women: 31.3, 45.2, 56.2 years). Similar comparability is seen for levels of the ergonomic index.

**Table 1:** Descriptive baseline characteristics at start of the first follow-up period

	Men				Women	
				
	18–39 years	40–49 years	50–64 years	18–39 years	40–49 years	50–64 years
					
	N (%)	N (%)	N (%)	N (%)	N (%)	N (%)
Total	5701 (30)	5248 (28)	7945 (42)	8749 (32)	7828 (29)	10 698 (39)
Physical work demand						
Low	1838 (32)	1929 (37)	2651 (33)	2443 (28)	2786 (36)	3389 (32)
Moderate	1334 (23)	1444 (28)	2379 (30)	2028 (23)	2159 (28)	3190 (30)
High	686 (12)	704 (13)	1189 (15)	1335 (15)	1142 (15)	1759 (16)
Very high	1195 (21)	894 (17)	1303 (16)	1771 (20)	1277 (16)	1763 (16)
Not available	648 (11)	277 (5)	423 (5)	1172 (13)	464 (6)	597 (6)
Working time						
Full-time	4589 (80)	4773 (91)	7129 (90)	4939 (56)	5078 (65)	6576 (61)
Part-time	801 (14)	327 (6)	533 (7)	2949 (34)	2470 (32)	3683 (34)
Not available	311 (5)	148 (3)	283 (4)	861 (10)	280 (4)	439 (4)
Body mass index						
Underweight	32 (1)	9 (0)	18 (0)	232 (3)	105 (1)	160 (1)
Normal	2667 (47)	1942 (37)	2696 (34)	4773 (55)	4111 (53)	5501 (51)
Overweight	1751 (31)	2244 (43)	3622 (46)	1590 (18)	2090 (27)	3032 (28)
Obesity	572 (10)	795 (15)	1246 (16)	830 (9)	1068 (14)	1424 (13)
Not available	679 (12)	258 (5)	363 (5)	1324 (15)	454 (6)	581 (5)
Smoking						
Non-smoker	3930 (69)	4027 (77)	6026 (76)	6186 (71)	6094 (78)	8215 (77)
Smoker	1168 (20)	979 (19)	1606 (20)	1462 (17)	1365 (17)	2028 (19)
Not available	603 (11)	242 (5)	313 (4)	1101 (13)	369 (5)	455 (4)
Physical activity						
No	3266 (57)	2887 (55)	4603 (58)	5398 (62)	4687 (60)	6474 (61)
Yes	2435 (43)	2361 (45)	3342 (42)	3351 (38)	3141 (40)	4224 (39)
Depression symptoms						
No	3793 (67)	3899 (74)	6210 (78)	5116 (58)	5461 (70)	7705 (72)
Yes	1300 (23)	1109 (21)	1414 (18)	2527 (29)	1997 (26)	2544 (24)
Not available	608 (11)	240 (5)	321 (4)	1106 (13)	370 (5)	449 (4)
Disease treatment						
No	4623 (81)	3951 (75)	5360 (67)	6442 (74)	5266 (67)	6911 (65)
Yes	1078 (19)	1297 (25)	2585 (33)	2307 (26)	2562 (33)	3787 (35)
Employment sector						
Private	3369 (59)	3468 (66)	4601 (58)	2560 (29)	2308 (29)	2506 (23)
Public	2332 (41)	1780 (34)	3344 (42)	6189 (71)	5520 (71)	8192 (77)
Highest educational level						
Short	648 (11)	603 (11)	1291 (16)	626 (7)	552 (7)	1508 (14)
Medium	2368 (42)	2223 (42)	3590 (45)	3147 (36)	3053 (39)	4198 (39)
Long	2665 (47)	2392 (46)	3004 (38)	4951 (57)	4208 (54)	4957 (46)
Not available	20 (0)	30 (1)	60 (1)	25 (0)	15 (0)	35 (0)
Number of survey waves						
1 of 3	4651 (82)	4076 (78)	6080 (77)	7083 (81)	6010 (77)	8174 (76)
2 of 3	491 (9)	436 (8)	536 (7)	814 (9)	683 (9)	722 (7)
3 of 3	559 (10)	736 (14)	1329 (17)	852 (10)	1135 (14)	1802 (17)

**Figure 1:**
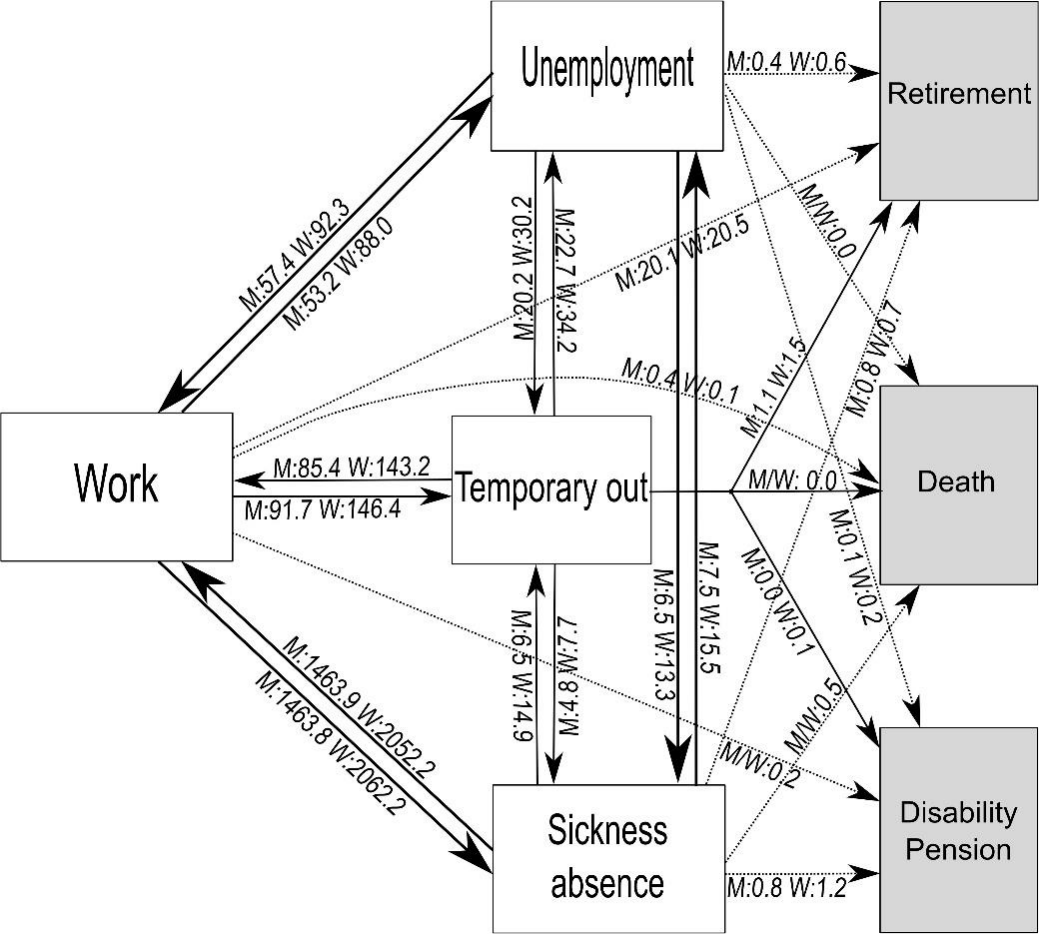
The Multi-state model with boxes as states and arrow as transitions including the number of transitions per 1000 person-years during follow-up for men (M) and women (W).

Figure 1 shows that during the follow-up period, the transitions between the work and the sickness absence states were the most frequent, with the highest transition incidence for women. A high transition incidence for women is moreover seen for the transitions between work and unemployment, though not as frequent as between work and sickness absence, and between work and temporary out, when compared to the respective transitions incidences of the men.

Table 2 shows that for men, the risk of a transition to sickness absence from work increases with an increasing level of physical work demands – except for young men with moderate and high physical work demands. The highest risk is seen for men aged 40–49 years having very high physical work demands (48%). A similar pattern is not seen for the women, but both men and women aged ≥40 years have lover likelihood of returning to work from sickness absence if they experienced high and very high physical work demands. The risk of being unemployed is highly associated with high or very high physical work demands – only a moderately equivalent likelihood is seen for a transition back to work.

**Table 2:** Hazard ratios (HR) and 95% confidence intervals (CI) for the transitions between the states of work, sickness absence, and unemployment state.

Physical work demand	Men			Women		
	
	18-39 years	40-49 years	50-64 years	18-39 years	40-49 years	50-64 years
					
	HR (95% CI)	HR (95% CI)	HR (95% CI)	HR (95% CI)	HR (95% CI)	HR (95% CI)
Work to sickness absence						
Low	1.00 (-)	1.00 (-)	1.00 (-)	1.00 (-)	1.00 (-)	1.00 (-)
Moderate	0.95 (0.83-1.10)	1.14 (1.00-1.30)	1.11 (1.00-1.24)	0.96 (0.87-1.05)	1.08 (0.99-1.16)	1.04 (0.96-1.13)
High	0.94 (0.76-1.16)	1.37 (1.14-1.65)^a^	1.28 (1.13-1.46)^a^	1.10 (1.00-1.22)	1.18 (1.06-1.32)^a^	1.03 (0.95-1.12)
Very high	1.25 (1.08-1.45)^a^	1.48 (1.30-1.70)^a^	1.36 (1.19-1.55)^a^	0.96 (0.81-1.13)	1.10 (0.99-1.22)	1.05 (0.96-1.15)
Work to Unemployment						
Low	1.00 (-)	1.00 (-)	1.00 (-)	1.00 (-)	1.00 (-)	1.00 (-)
Moderate	1.92 (1.02-3.63)^b^	1.91 (0.87-4.20)	1.76 (0.82-3.77)	1.54 (1.04-2.28)^b^	1.63 (1.00-2.68)	1.32 (0.88-1.97)
High	1.77 (0.94-3.31)	7.41 (2.30-23.83)^a^	2.63 (1.44-4.80)^a^	1.83 (1.14-2.95)^b^	1.97 (1.14-3.41)^b^	2.24 (1.45-3.45)^a^
Very high	3.56 (1.97-6.43)^a^	3.45 (1.54-7.74)^a^	5.37 (2.22-12.96)^a^	2.72 (1.53-4.84)^a^	12.23 (2.64-56.62)^a^	3.16 (1.83-5.45)^a^
Sickness absence to Work						
Low	1.00 (-)	1.00 (-)	1.00 (-)	1.00 (-)	1.00 (-)	1.00 (-)
Moderate	1.17 (0.84-1.63)	0.75 (0.54-1.03)	1.01 (0.77-1.34)	0.98 (0.80-1.22)	0.78 (0.66-0.93)^a^	0.74 (0.60-0.91)^a^
High	0.89 (0.62-1.30)	0.53 (0.35-0.81)^a^	0.65 (0.45-0.93)^b^	0.94 (0.75-1.18)	0.74 (0.60-0.91)^a^	0.70 (0.57-0.85)^a^
Very high	0.73 (0.49-1.08)	0.49 (0.38-0.64)^a^	0.84 (0.63-1.13)	0.98 (0.80-1.19)	0.56 (0.41-0.76)^a^	0.60 (0.50-0.71)^a^
Sickness absence to unemployment					
Low	1.00 (-)	1.00 (-)	1.00 (-)	1.00 (-)	1.00 (-)	1.00 (-)
Moderate	1.06 (0.38-3.00)	1.66 (0.36-7.63)	0.87 (0.36-2.12)	2.12 (0.95-4.71)	0.92 (0.53-1.60)	2.20 (0.92-5.27)
High	2.57 (0.88-7.47)	0.00 (0.00-0.02)^a^	0.85 (0.26-2.73)	1.33 (0.63-2.83)	0.65 (0.34-1.26)	0.86 (0.44-1.71)
Very high	2.84 (0.94-8.57)	0.66 (0.16-2.75)	3.79 (1.45-9.90)^a^	2.32 (1.01-5.29)^b^	0.96 (0.51-1.82)	1.32 (0.73-2.36)
Unemployment to Work						
Low	1.00 (-)	1.00 (-)	1.00 (-)	1.00 (-)	1.00 (-)	1.00 (-)
Moderate	1.46 (0.80-2.67)	2.34 (1.02-5.37)^b^	1.84 (1.08-3.16)^b^	1.36 (0.92-1.99)	1.35 (0.86-2.14)	1.04 (0.65-1.66)
High	1.15 (0.56-2.37)	2.34 (0.90-6.10)	1.12 (0.57-2.21)	1.46 (0.91-2.35)	1.81 (1.13-2.91)^b^	1.41 (0.86-2.31)
Very high	1.47 (0.97-2.23)	2.55 (1.09-5.94)^b^	1.49 (0.85-2.61)	1.69 (1.09-2.61)^b^	2.97 (1.78-4.97)^a^	2.10 (1.32-3.35)^a^
Unemployment to Sickness absence					
Low	1.00 (-)	1.00 (-)	1.00 (-)	1.00 (-)	1.00 (-)	1.00 (-)
Moderate	1.32 (0.47-3.73)	2.80 (0.53-14.82)	0.77 (0.30-2.00)	1.86 (0.95-3.62)	1.05 (0.58-1.90)	2.00 (1.09-3.68)^b^
High	1.35 (0.37-4.87)	2.43 (0.37-16.08)	1.29 (0.29-5.70)	1.53 (0.79-2.99)	0.92 (0.46-1.83)	0.75 (0.38-1.48)
Very high	0.86 (0.33-2.29)	1.33 (0.22-8.11)	2.48 (1.04-5.87)^b^	1.53 (0.75-3.13)	0.72 (0.23-2.28)	1.23 (0.73-2.07)

a 1% significant.

b 5% significant

Supplementary material table C1 shows – in supplement to figure 1 and table 2 – the raw number of transitions (events) occurring between work, sickness absence, and unemployment along with the unadjusted transition incidences by the number of events per 1000 person-years.

**Figure 2:**
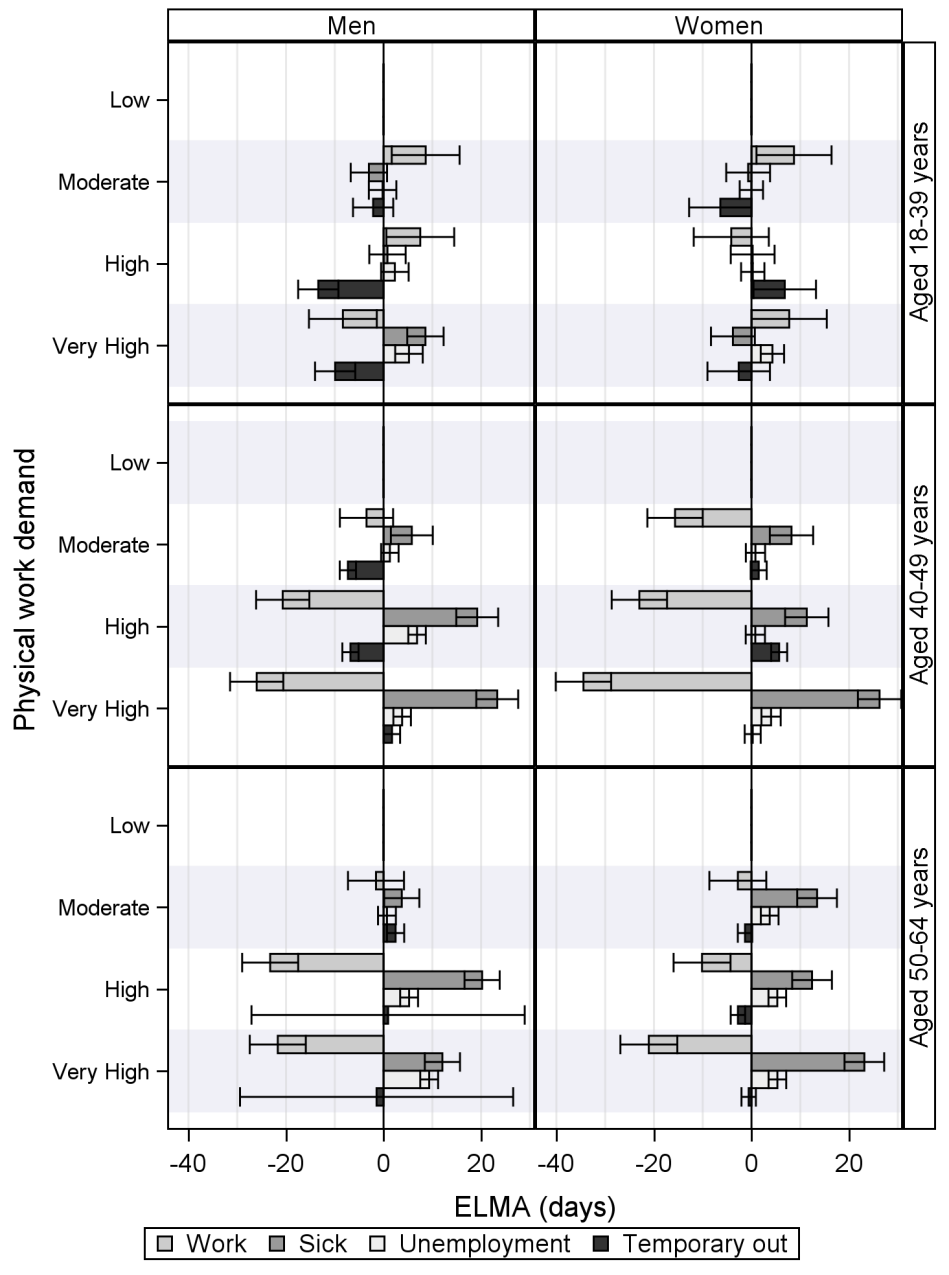
The expected labor market affiliation (ELMA) results by the expected duration (+/-) of working time, sickness absence, unemployment, and temporarily out (of 730 days) when compared to the absolute duration time of individuals with low physical work demands. Grouped by gender and age.

Figure 2 shows that the additional expected time in work, sickness absence, and unemployment for young men and women is mostly unaffected by physical work demands. However, a steep increased in sickness absence time is seen for the middle-aged men (6, 19, and 23 days respectively) and women (8, 11, and 26 days respectively) by increasing physical work demands with a parallel decline in working time (table 3 presents the precise estimates).

**Table 3:** The expected labor market affiliation (ELMA) and crude mean results of the expected change (+/-) in duration of working time, sickness absence, unemployment, and temporarily out (per 730 days) when compared to the absolute duration time of individuals with low level of physical work demands (reference group). Grouped by gender and age. Supplementary tables (1B), shows the additional results of the three absorbing states. [CI=confidence interval.]

Physical work demand	Work		Sickness absence		Unemployment		Temporary Out	
			
	ELMA	Crude	ELMA	Crude	ELMA	Crude	ELMA	Crude
							
	days (95% CI)	days	days (95% CI)	days	days (95% CI)	days	days (95% CI)	days
Men								
18-39 years								
Low (reference)	677.1 (672.2:682.0)^a^	582.9	14.6 (12.0:17.3)^a^	9.3	5.4 (3.4:7.4)^a^	5.4	31.2 (28.3:34.1)^a^	23.6
Moderate	+ 8.6 (1.7:15.6)b	+ 14.0	- 3.0 (-6.7:0.7)	+ 2.2	- 0.2 (-3.0:2.6)	+ 2.3	- 2.1 (-6.3:2.0)	+ 5.4
High	+ 7.5 (0.6:14.5)b	- 33.9	+ 0.8 (-2.9:4.5)	+ 5.5	+ 2.3 (-0.5:5.1)	+ 3.3	- 13.4 (-17.5:-9.3)^a^	+ 4.3
Very high	- 8.3 (-15.3:-1.4)b	- 80.7	+ 8.6 (4.9:12.3)^a^	+ 9.0	+ 5.2 (2.4:8.0)^a^	+ 7.8	- 9.9 (-14.1:-5.8)^a^	- 0.7
40-49 years								
Low (reference)	704.8 (700.9:708.6)^a^	602.0	9.6 (6.6:12.7)^a^	9.2	2.8 (1.6:4.1)^a^	1.8	9.9 (8.7:11.1)^a^	6.0
Moderate	- 3.5 (-9.0:2.0)	+ 13.8	+ 5.8 (1.5:10.1)^a^	+ 5.2	+ 1.3 (-0.5:3.1)	+ 2.1	- 7.3 (-9.0:-5.6)^a^	- 2.7
High	- 20.7 (-26.1:-15.2)^a^	- 28.0	+ 19.2 (14.9:23.5)^a^	+ 9.5	+ 6.9 (5.1:8.7)^a^	+ 4.1	- 6.8 (-8.5:-5.1)^a^	- 2.7
Very high	- 26.0 (-31.5:-20.6)^a^	- 79.0	+ 23.3 (19.0:27.6)^a^	+ 13.3	+ 3.8 (2.0:5.6)^a^	+ 3.9	+ 1.7 (0.1:3.4)^b^	+ 0.8
50–64 years								
Low (reference)	671.0 (666.9:675.0)^a^	587.5	18.5 (15.9:21.0)^a^	15.1	3.6 (2.3:4.9)^a^	3.5	4.8 (3.5:6.0)^a^	5.3
Moderate	- 1.6 (-7.3:4.2)	- 1.3	+ 3.7 (0.1:7.3)^b^	+ 2.5	+ 0.7 (-1.1:2.5)	+ 0.6	- 0.0 (-0.2:0.2)	+ 1.5
High	- 23.2 (-29.0:-17.5)^a^	- 30.6	+ 20.2 (16.6:23.8)^a^	+ 10.6	+ 5.2 (3.4:7.1)^a^	+ 4.5	+ 0.7 (0.5:0.9)^a^	- 0.2
Very high	- 21.7 (-27.4:-15.9)^a^	- 58.9	+ 12.1 (8.5:15.7)^a^	+ 13.1	+ 9.4 (7.5:11.2)^a^	+ 8.9	+ 0.5 (0.3:0.7)^a^	- 0.8
Women								
18-39 years								
Low (reference)	616.5 (611.1:622.0)^a^	568.6	32.8 (29.6:36.0)^a^	23.7	9.2 (7.5:10.9)^a^	7.9	61.5 (57.0:66.1)^a^	61.0
Moderate	+ 8.7 (1.0:16.4)^b^	+ 21.3	- 0.7 (-5.2:3.8)	+ 4.0	- 0.0 (-2.4:2.4)	+ 1.9	- 6.4 (-12.8:0.0)	+ 3.2
High	- 4.1 (-11.8:3.5)	- 3.1	+ 0.2 (-4.3:4.7)	+ 8.8	+ 0.3 (-2.1:2.7)	+ 3.3	+ 6.8 (0.4:13.2)^b^	+ 5.4
Very high	+ 7.7 (0.0:15.4)^b^	- 24.8	- 3.8 (-8.3:0.7)	+ 9.3	+ 4.3 (1.9:6.7)^a^	+ 6.2	- 2.6 (-9.0:3.8)	- 0.8
40-49 years								
Low (reference)	688.7 (684.7:692.7)^a^	628.4	27.2 (24.1:30.3)^a^	23.8	6.0 (4.6:7.4)^a^	5.3	6.3 (5.2:7.5)^a^	6.9
Moderate	- 15.7 (-21.4:-10.0)^a^	+ 17.1	+ 8.2 (3.8:12.6)^a^	+ 10.9	+ 0.8 (-1.2:2.8)	+ 1.3	+ 1.5 (-0.2:3.1)	+ 0.2
High	- 23.0 (-28.7:-17.3)^a^	+ 12.8	+ 11.3 (6.9:15.8)^a^	+ 15.5	+ 0.8 (-1.2:2.8)	+ 2.6	+ 5.7 (4.0:7.3)^a^	+ 2.1
Very high	- 34.5 (-40.1:-28.8)^a^	- 18.3	+ 26.3 (21.8:30.7)^a^	+ 22.3	+ 4.0 (2.0:6.0)^a^	+ 7.4	+ 0.2 (-1.4:1.9)	+ 0.1
50–64 years								
Low (reference)	654.1 (650.0:658.3)^a^	609.3	27.8 (24.9:30.7)^a^	24.7	5.0 (3.7:6.3)^a^	5.2	5.6 (4.5:6.6)^a^	5.4
Moderate	- 2.8 (-8.6:3.0)	+ 10.8	+ 13.4 (9.4:17.5)^a^	+ 7.9	+ 3.7 (1.9:5.5)^a^	+ 2.3	- 1.3 (-2.8:0.1)	- 0.9
High	- 10.1 (-16.0:-4.3)^a^	+ 7.7	+ 12.4 (8.3:16.5)^a^	+ 12.1	+ 5.3 (3.5:7.1)^a^	+ 3.7	- 2.8 (-4.3:-1.3)^a^	- 2.6
Very high	- 21.1 (-26.9:-15.2)^a^	- 25.2	+ 23.1 (19.1:27.2)^a^	+ 23.2	+ 5.3 (3.5:7.1)^a^	+ 5.5	- 0.6 (-2.1:0.9)	+ 0.1

a 1% significant.

b 5% significant

For the oldest age group, the effect of physical demand level is more complex. Women with moderate and high physical work demands experience an almost identical increase of sickness absence time (12 and 13 days, respectively), while women with very high physical work demands experience additionally 23 sickness absence days – compared to 28 for women with low physical work demands.

Moderate physical work demands have almost no effect on the men aged 50–64 years. However, a high level inflicts additionally 20 days of sickness absence, while a very high level inflicts 12 additional days of sickness absence and 9 additional days of unemployment. The time in the ’temporary out’ state is highly uncertain for men with high and very high physical work demands.

For men and women aged 50–64 years, one can calculate expected time spend in retirement (supplementary table 1D). However, for those having moderate, high and very high physical work demands, the results show a small decline in retirement time – most for men having a high level (10 days) and next for women with very high physical work demands (8 days).

Generally, we found fair agreement between the ELMA and the crude estimates. However, across working time outcomes the ELMA method found higher numbers in the reference group than the crude estimates.

## Discussion

In this prospective longitudinal study, we showed that physically demanding work is associated with poorer labor market affiliation of Danish employees. Physical work demands were measured using a combined ergonomic index and categorized into four levels. The study used several highly detailed registers with date-based information and included all lengths of sickness absence of both public and private employees.

By using the ELMA method, we showed that, moderate-to-very high physical work demands were associated with increased sickness absence time and decreased time working, but only for employees ≥40 years. For the younger employees, physical work demands did not affect labor market affiliation within the two-year follow-up period. These findings agree with previous findings showing increased risk of long-term sickness absence in older – but not younger – workers from high physical work demands (1). There may be several reasons for these findings. First, as muscle strength declines with increasing age (23), younger workers are better physically fit for the job than older workers. Thus, at any given absolute workload, the relative workload is lower among younger workers. This may also have consequences for muscle recovery after work. Second, the accumulated hazardous effect of high physical work demands is more likely to affect older workers because they have been exposed for more years (ie, a higher accumulated exposure time) (24).

The decrease in time working and increased sickness absence time are significant from moderate physical work demands, but most pronounced for men and women with high and very high physical work demands. However, the effect for men with a moderate level was very low. The higher level of muscle strength among men compared with women (23) may explain that moderate levels of physical work demands only affected men to a minor extent. Another possible explanation is the somewhat gender-segregated labor market in Denmark, ie, different types of physical work is performed by men and women, for example, cleaning, hospital and elderly care occupations are more frequent among women, while more men are occupied as bricklayers, carpenters, or in similar jobs (25).

### Comparison with previous studies

Concerning the risk of a transition from work to sickness absence, our results are in line with a previous analysis using traditional Cox-regression analyses and the same ergonomic index (1). We found comparably increased risks of sickness absence for employees aged ≥40 years with increasing physical work demands. However, our results show that this almost linear increasing effect is most pronounced among men, whereas this is not seen among women. For the likelihood of returning to work from sickness absence, we found similar results for both genders as the risk reduced with increasing physical work demands. These results may be due to the use of all length sickness absence instead of solely long-term sickness absence (11) and that we included all employees regardless of prior sickness absence.

Only a sparse number of previous studies use multi-state modeling for investigating labor market affiliation, and even fewer focus on physical work demands as exposure (10, 26, 27), which limits the comparison with previous results. Pedersen et al (9) used a life course perspective and found comparable decreased working-time and increased sickness absence and unemployment time for employees aged >40 years with high physical work demands.

The results suggest that special attention should be paid to middle-age and older employees in occupations characterized by high or very high physical work demands. For this age group, our results suggest a potential for decreasing the time in sickness absence and increasing the working-time if the level of physical demand is lowered. A potential gain in effective working-time is additionally present for women with a moderate level of physically demanding work.

### Strengths and limitations

The study strengths include a substantial sample of Danish employees from three survey waves. The study analyses the individual labor market affiliation on a day-to-day basis, by a linkage with detailed register data. The study incorporates a multi-level and -state setting controlling for recurrent and competing events. Moreover, the study includes sickness absence periods down to a duration of one day. The data and information retained from the surveys and registers all contained a high level of consistency during the entire follow-up period (2012–2018).

The flexibility of the ELMA method makes it possible to examine different aspects of the labor market affiliation and to include time-dependent variables and weights. Compared to a crude mean of state-specific duration time, this adds important new knowledge on transitions changes in labor market affiliation on individuals having physically demanding jobs. The ELMA method is also effective in highlighting trends in labor marked outcomes not easily identified from the HR estimated in the multi-state models. For example, for women in the 50–64 year age group, a clear and statistically highly significant increase in sick days is seen with increased physical work demands (table 3). This trend is not immediately clear from the HR in table 2 that do not show a significant increased risk of going from work to sickness absence for 50–64-year-old women with high physical work demands. The increase in sick days seen in table 3 is due to several factors but mainly to a much lower chance of getting back to work from sickness absence for middle-aged women with high physical demands (table 2). This example illustrated the ability of the ELMA method to summarize complicated results. Moreover, comparing ELMA with the crude results reflects the fact that the ELMA method presents the expected labor market outcomes. This means handling individuals that are censored and time when not at risk better than the crude estimates, which are highly affected by censoring and time when not at risk, implying generally lower risk estimates.

The use of multiple survey waves increases the total sample size and adds the possibility of incorporating time-dependent adjustment of the exposure to the analysis. This is possible as the baselines of the two-year follow-up are set individually thoughout 2012 and 2016, and set repeatedly for employees attending multiple survey waves. A large sample strengthens the multi-state analysis as multiple transitions between the states are likely to occur and increase the group sizes, eg the number of employees having a very high level of physical work exposure. However, the use of the relatively short two-year follow-up period between the survey waves implies limitations concerning the long-term perspective of the individual labor market affiliation. For example, is the number of employees experiencing long-term effects of physical work demands underestimated eg, employees experiencing disability pension? This is because individuals cannot attend the survey if they are no longer employed, but instead are on, for example, long-term sickness absence or unemployment benefit while awaiting disability pension.

The study includes additional limitations. First, the sample represents a wide variety of Danish employees and the study is likely to be generalizable to the Danish workforce, however, some caution should be taken due to lack of response from men and young employees (WEHD) (11, 12) and the limits of RoWA concerning small private companies (14, 15). Second, though only a few individuals entered the disability pension and pension state, there is a small possibility of overestimating the time spend there as the model did not include the possibility of a transition from disability pension to pension, from disability pension to death, and from pension to death. Third, the study included both part-time and full-time benefits and, if multiple benefits were paid simultaneously or along with salary payments, a prioritization between payments was made. This is likely to slightly underestimate the duration of the working time and overestimate the duration of the other states. Fourth, the study relates to the Danish labor market system, which makes comparison with other countries difficult. However, the results should make room for some general consideration on employees experiencing physically demanding jobs. Finally, the study does not include all aspects of the physical workload, it is for example likely that chronic disease and other health-related conditions will influence the working-time and time with sickness absence.

### Concluding remarks

Moderate-to-very high levels of physical work demands are associated with markedly reduced active labor market affiliation among middle-aged and older Danish employees but not among young workers. The changes of the expected labor market affiliation mainly concern increased time in sickness absence on the cost of reduced active working-time. Preventive initiatives focusing on gender and age of the employees are likely to decrease the negative impact of physically demanding work in occupations with high physical work exposures.

## Supplementary material

Supplementary material

## Data sharing statement

The SAS code can be shared upon reasonable request by authorized researchers after application to the NRCWE. Data is available on the Researcher access at Statistics Denmark, see www.dst.dk/en/TilSalg/Forskningsservice.
