# Supplementary material for: Physical work demands and expected labor market affiliation (ELMA): Prospective cohort with register-follow-up among 46 169 employees
Source: Scand J Work Environ Health. 2022 Oct 29;48(8):641–50. doi: 10.5271/sjweh.4050 (PMC10546615; doi:10.5271/sjweh.4050)
Supplement: Supplementary material [file SJWEH-48-641-S001.pdf]

Physical work demands and expected labor market affiliation (ELMA): Prospective cohort with register-follow-up among 46 169 employees[1]

By Jacob Pedersen, PhD,[2] Jakob Bue Bjorner, PhD, Lars L Andersen, PhD

1. Supplementary material
2. Correspondence to: Jacob Pedersen, National Research Centre for the Working Environment Lersø Parkallé 105. DK-2100 Copenhagen Ø, Denmark. [jpe@nfa.dk]

A) Questions for physical index:

'How much of your working time do you …' (1) walk or stand? (2) work with twisted or bent back without support from the hands and arms? (3) have the arms lifted to or above shoulder height, (4) do the same arm movements several times a minute? (e.g., package work, mounting, machine feeding, carving), (5) squat or kneel when you work? (6) push or pull?, and (7) lift or carry? The seven questionnaires all contained the same response range with the corresponding index score: 'almost all the time' score 100, 'approx. 3/4 of the time' score 75, 'approx. 1/2 of the time' score 50, 'approx. 1/4 of the time' score 25, 'seldom/very little' score 12.5, and 'never' score 0.

B) Jurisdictional context: the Danish labor market

The Danish labor market is characterized as a flexicurity system with generally high labor market participation rates (73% for females and 77% for males) (Statistics Denmark 2019), low formal employment protection inflicting a high turnover of the workforce. The "security" of the flexicurity contains generous and accessible social benefits on e.g. sickness absence, unemployment, and disability pension (Madsen 2005). Sickness absence benefit are typically paid from 30 days of continuous absence as a compensation to the employer for paying salary during the sickness absence period. Two types of unemployment benefits exists - one based on an insurance scheme and one that is accessible to all. Disability pension is available to all with a major disability that limits the workability.

The official retirement age in Denmark is rising but was 65 years during the study follow-up period. It is possible to retire at an earlier age; the most common of such possibilities is the Voluntary Early retirement scheme that is accessible to all employees and makes it possible to retire up to five years ahead of the official retirement age. However, for the scheme to become an option, one must have contributed continuously to the scheme for a minimum of 30 years. One may additionally retire early by the use of own savings.

C) Table 1C. Number of transitions (events) and events per 1000 person-years during follow-up between the states work, sickness absence, and unemployment. Divided by gender, age group, transition, and group of physical index.

Transition	Physical work demand	Men 18-39 years Ev(n)	Men 18-39 years Ev(n)/10³PY	Men 40-49 years Ev(n)	Men 40-49 years Ev(n)/10³PY	Men 50-64 years Ev(n)	Men 50-64 years Ev(n)/10³PY	Women 18-39 years Ev(n)	Women 18-39 years Ev(n)/10³PY	Women 40-49 years Ev(n)	Women 40-49 years Ev(n)/10³PY	Women 50-64 years Ev(n)	Women 50-64 years Ev(n)/10³PY
Work to Sickness absence													
	Low	5302	1380,8	5339	1239,9	7926	1304,6	11107	2049,0	13167	1929,6	15621	1876,0
	Moderate	4450	1576,6	4993	1534,5	7670	1427,7	9971	2129,1	12237	2217,6	16718	2073,1
	High	2043	1580,1	2503	1725,0	4108	1607,3	6982	2367,2	6605	2288,0	9323	2065,4
	Very high	3267	1598,7	3168	1883,6	4721	1765,7	7996	2208,1	6781	2338,2	9203	2160,9
Work to Unemployment													
	Low	122	31,8	38	8,8	138	22,7	317	58,5	225	33,0	313	37,6
	Moderate	190	67,3	125	38,4	200	37,2	455	97,2	308	55,8	488	60,5
	High	91	70,4	84	57,9	163	63,8	309	104,8	218	75,5	439	97,3
	Very high	189	92,5	104	61,8	236	88,3	461	127,3	330	113,8	462	108,5
Sickness absence to Work													
	Low	5318	1385	5355	1243,6	7933	1305,8	11029	2034,6	13148	1926,8	15585	1871,7
	Moderate	4446	1575,1	4979	1530,2	7667	1427,2	9907	2115,5	12200	2210,9	16646	2064,2
	High	2035	1573,9	2504	1725,6	4106	1606,5	6908	2342,1	6580	2279,3	9285	2057
	Very high	3263	1596,7	3169	1884,2	4704	1759,4	7896	2180,5	6750	2327,5	9166	2152,2
Sickness absence to Unemployment													
	Low	15	3,9	6	1,4	26	4,3	66	12,2	50	7,3	37	4,4
	Moderate	14	5,0	16	4,9	21	3,9	78	16,7	55	10,0	86	10,7
	High	15	11,6	<5	0,7	13	5,1	68	23,1	29	10,0	36	8,0
	Very high	28	13,7	7	4,2	59	22,1	87	24,0	55	19,0	63	14,8
Unemployment to Work													
	Low	136	35,4	46	10,7	138	22,7	327	60,3	230	33,7	312	37,5
	Moderate	186	65,9	137	42,1	199	37,0	462	98,7	304	55,1	479	59,4
	High	99	76,6	78	53,8	168	65,7	307	104,1	234	81,1	428	94,8
	Very high	210	102,8	114	67,8	244	91,3	472	130,3	345	119	476	111,8
Unemployment to Sickness absence													
	Low	12	3,1	6	1,4	26	4,3	60	11,1	49	7,2	31	3,7
	Moderate	16	5,7	15	4,6	15	2,8	73	15,6	51	9,2	75	9,3
	High	9	7,0	<5	1,4	11	4,3	60	20,3	26	9,0	28	6,2
	Very high	15	7,3	<5	2,4	56	20,9	74	20,4	43	14,8	51	12,0

Ev(n): Events(n). Ev(n)/10^3PY: Events(n) per 1000 person-years

D) Table 1D. The ELMA and Crude mean results (in days incl. 95% confidence interval) of the expected change (+/-) in duration of disability pension, Pension, and death when compared to the absolute duration time of individuals with low level of physical demands (reference). Grouped by gender and age.

**Gender**	**Age**	**Physical work demand**	**Disability Pension**		**Pension**		**Death**	
			**ELMA days (95% CI)**	**Crude days**	**ELMA days (95% CI)**	**Crude days**	**ELMA days (95% CI)**	**Crude days**
Men	18-39 years	Low (ref.)	0.1 (0.1:0.2)†	0.1	-	-	-	-
		Moderate	-0.0 (-0.1:0.0)	+ 0.4	-	-	-	-
		High	-0.1 (-0.2:-0.1)†	-	-	-	-	-
		Very high	-0.1 (-0.2:-0.1)†	' 0.3	-	-	-	-
	40-49 years	Low (ref.)	0.1 (-0.0:0.2)	0.1	-	-	-	-
		Moderate	- 0.1 (-0.2:0.1)	-	-	-	-	-
		High	+ 0.2 (0.1:0.3)†	+ 0.5	-	-	-	-
		Very high	+ 0.1 (-0.0:0.2)	+ 0.2	-	-	-	-
	50-64 years	Low (ref.)	0.2 (0.0:0.3)	0.1	33.1 (31.1:35.1)†	30	0.3 (0.2:0.5)†	0.4
		Moderate	- 0.0 (-0.2:0.2)	+ 0.3	- 7.1 (-9.9:-4.3)†	+ 3.9	+ 0.5 (0.2:0.7)†	+ 0.6
		High	+ 0.7 (0.5:0.9)†	+ 0.9	- 9.6 (-12.4:-6.8)†	- 2.9	+ 0.3 (0.1:0.6)†	+ 0.3
		Very high	+ 0.5 (0.3:0.7)†	+ 0.5	- 4.3 (-7.1:-1.4)†	- 4.7	+ 0.3 (0.1:0.6)†	+ 0.4
Women	18-39 years	Low	0.2 (0.2:0.3)†	0.3	-	-	-	-
		Moderate	0.2 (-0.3:0.1)†	- 0.1	-	-	-	-
		High	0.2 (-0.3:0.1)†	0.0	-	-	-	-
		Very high	0.2 (-0.3:0.1)†	- 0.2	-	-	-	-
	40-49 years	Low	0.1 (0.1:0.1)*	0.1	-	-	-	-
		Moderate	0.0 (-0.0:0.1)	+ 0.6	-	-	-	-
		High	+ 0.1 (0.1:0.2)†	+ 1.2	-	-	-	-
		Very high	- 0.1 (-0.1:-0.0)†	+ 0.1	-	-	-	-
	50-64 years	Low	1.0 (0.8:1.2)†	0.9	33.9 (32.0:35.8)†	32.4	0.4 (0.1:0.6)†	0.3
		Moderate	- 0.5 (-0.8:-0.2)†	- 0.4	- 5.0 (-7.7:-2.2)†	+ 4.9	- 0.1 (-0.4:0.2)	+ 0.1
		High	- 0.8 (-1.1:-0.5)†	+ 0.1	- 5.2 (-8.0:-2.5)†	+ 5.0	0.0 (-0.3:0.3)	+ 0.1
		Very high	- 0.8 (-1.1:-0.6)†	0.0	- 7.8 (-10.6:-5.1)†	+ 8.1	- 0.3 (-0.6:0.1)	+ 0.1

†: 1% significant. *: 5% significant. ELMA: Expected Labor Market Affiliation. Ref: Reference value.
